# Comparison of half-molar sodium lactate and mannitol to treat brain edema in severe traumatic brain injury: A systematic review

**DOI:** 10.1016/j.cjtee.2021.07.005

**Published:** 2021-07-13

**Authors:** Abdul Hafid Bajamal, Tedy Apriawan, I.G.M. Aswin R. Ranuh, Franco Servadei, Muhammad Faris, Asra Al Fauzi

**Affiliations:** a Department of Neurosurgery, Faculty of Medicine, Universitas Airlangga, Dr. Soetomo General Academic Hospital, Surabaya, Indonesia; b Department of Neurosurgery, Humanitas Clinical and Research Hospital, Humanitas University, Milan, Italy

**Keywords:** Half-molar sodium lactate, Mannitol, Traumatic brain injuries

## Abstract

**Purpose:**

Hypertonic fluids such as mannitol and half-molar sodium lactate are given to treat intracranial hypertension in patients with severe traumatic brain injury (TBI). In this study, sodium lactate was compared to mannitol in patients with TBI to investigate the efficacy in reducing intracranial pressure (ICP).

**Methods:**

This study was a systematic review with literature research on articles published in any year in the databases of PubMed, ScienceDirect, *Asian Journal of Neurosurgery*, and Cochrane Central Register of Controlled Trials. The keywords were “half-molar sodium lactate”, “mannitol”, “cerebral edema or brain swelling”, and “severe traumatic brain injury”. The inclusion criteria were (1) studies published in English, (2) randomized control trials or retrospective/prospective studies on TBI patients, and (3) therapies including half-molar sodium lactate and mannitol and (4) sufficient data such as mean difference (MD) and risk ratio (RR). Data analysis was conducted using Review Manager 5.3.

**Results:**

From 1499 studies, a total of 8 studies were eligible. Mannitol group reduced ICP of 0.65 times (MD 0.65; *p* = 0.64) and improved cerebral perfusion pressure of 0.61 times (MD 0.61; *p* = 0.88), better than the half-molar group of sodium lactate. But the half-molar group of sodium lactate maintained the mean arterial pressure level of 0.86 times, better than the mannitol group (MD 0.86; *p* = 0.09).

**Conclusion:**

Half-molar sodium lactate is as effective as mannitol in reducing ICP in the early phase of brain injury, superior over mannitol in an extended period. It is able to prevent intracranial hypertension and give better brain tissue perfusion as well as more stable hemodynamics. Blood osmolarity is a concern as it increases serum sodium.

## Introduction

Traumatic brain injury (TBI) is the global main cause of mortality among patients aged 5–35 years. Each year, around 1.5 million patients with TBI die and some other millions require emergency treatment.^1^ According to estimates from the World Health Organization, almost 90% of deaths due to injuries occur in middle-to-low-income countries, like Indonesia.^2^

Prolonged, refractory increase of the intracranial pressure (ICP) is a central factor that might worsen the neurologic outcome following TBI. Therefore, ICP control is crucial in optimizing cerebral perfusion pressure (CPP). Among several therapeutic modalities, hypertonic saline is routinely administered to treat increased ICP in patients with severe TBIs.^3^ Hypertonic solution like mannitol and half-molar sodium lactate utilize osmotic pressure gradient between plasma and brain tissue to excrete fluid out from the brain tissue into the intravascular lumen.^4^

Disruption of energy supply in TBI may seriously affect the neurologic outcome if treated incorrectly.^5^ In the early phase of TBI, brain tissue enters a hypermetabolic state and causes a sharp rise of the blood glucose level in order to adapt with the pathological conditions following TBI.^6^ If this hypermetabolic condition is not leveled out by adequate energy supply, brain starvation occurs, which induces poor prognosis.^7^ Previous studies mentioned that lactate could act as an alternate for energy supply in the early phase of TBI.^8^^,^^9^ Moreover besides alternative energy source, half-molar sodium lactate has another function,^10^ i.e. osmotherapy due to its hypertonic nature. In this systematic review, we compared the osmotherapy function of sodium lactate with mannitol for TBIs.

## Methods

### Information source and search strategy

Selection of studies was carried out with the Preferred Reporting Items for Systematic Review and Meta-Analysis Protocol method guideline.^11^ The data were collected from the databases of PubMed, ScienceDirect, *Asian Journal of Neurosurgery*, Cochrane Central Register of Controlled Trials, with identified relevant researches. We used the following keywords: “half-molar sodium lactate”, “mannitol”, “cerebral edema or brain swelling”, and “severe TBI”.

### Eligibility criteria

Inclusion criteria for this systematic review were the following: (1) published in English; (2) randomized controlled trial (RCT) or retrospective/prospective study of TBI patients who were treated with either half-molar sodium lactate or mannitol; and (3) enough data to calculate the mean difference (MD) and risk ratio (RR). Cohort, commentary column, newspaper, editorial, case report, and experts’ opinion were excluded. Studies that included non-TBI cases, pediatric patients, or those without quantitative data from primary outcome were also excluded.

### Article assessment

The chosen studies have been fully checked for names of the first author, publication year, study design, demographic data, Glasgow coma scale (GCS) score, presence of intracranial hypertension and ICP level, osmolality level, doses & administration formula of half-molar sodium lactate and mannitol, and outcomes.

Study characteristics are presented in the patient-intervention-comparison-outcome table (Table 1). Quality of each study was assessed using the Risk-of-bias Assessment Tool Based on The Cochrane Handbook for Systematic Reviews of Interventions (version 5.3.0).^12^

**Table 1:** Patient-intervention-comparison-outcome table.

Patient	Patients aged 15–100 years, with severe TBI, either operated or not
Intervention	Half-molar sodium lactate
Comparison/Control	Mannitol
Outcome	Level of intracranial pressure, mean arterial blood pressure, cerebral perfusion pressure, sodium serum, serum osmolality, blood glucose

### Outcome variables

The analyzed variables were mean decreases of ICP, mean arterial blood pressure (MABP), CPP, sodium serum, serum osmolality, and blood glucose. Diagnostic criteria used in each study could differ to each other. Revman (version 5.4) from the Cochrane Review was used for statistical analysis.

## Results

### Study selection, characteristics, and quality assessment

**Fig. 1:**
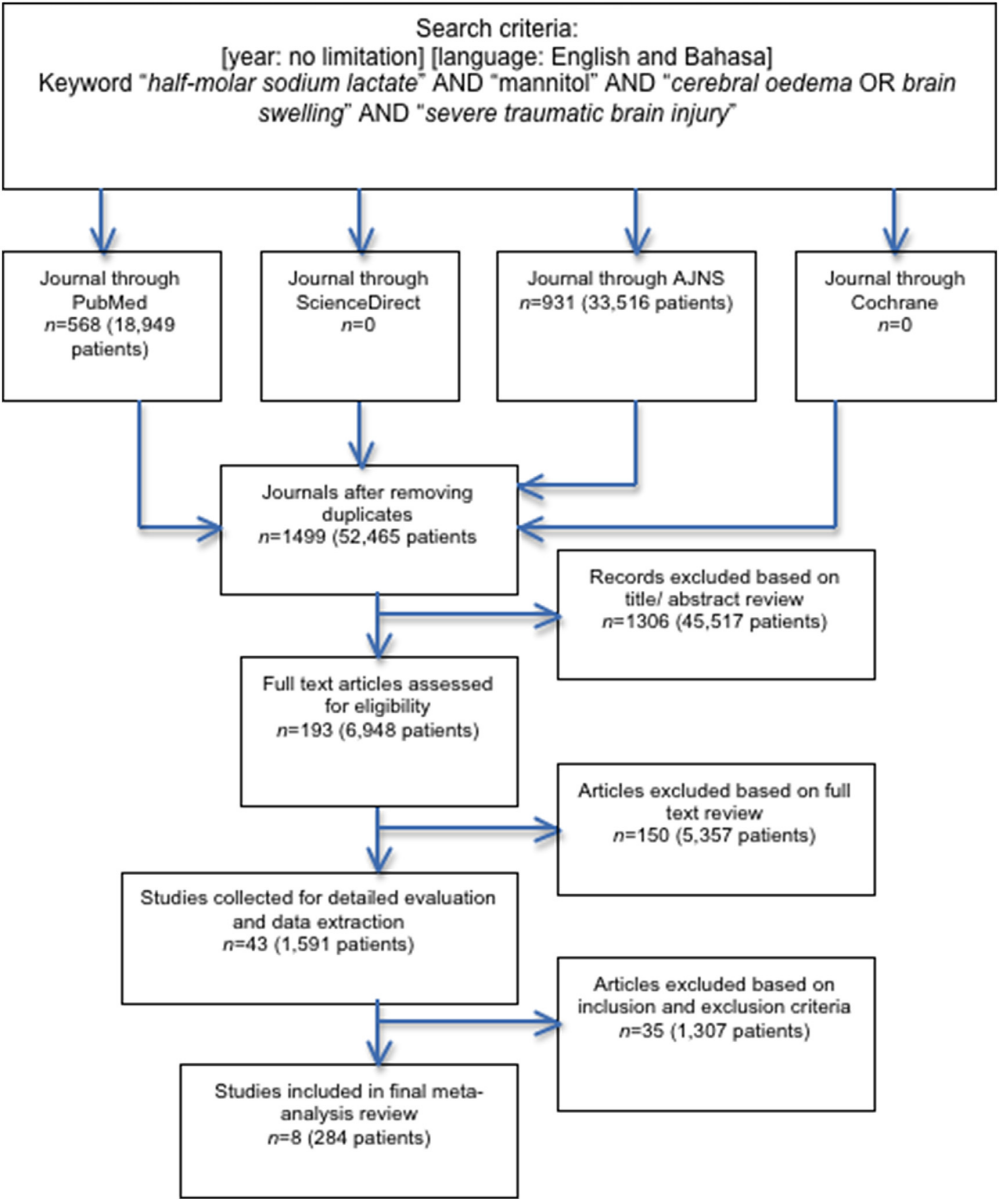
Study flow chart.

The flow chart of the research selection can be seen in Fig. 1. We obtained 1499 abstracts of articles after a search for topics about “half-molar sodium lactate”, “mannitol”, “cerebral edema or brain swelling”, and “severe TBI”. A total of 8 articles were included in this study after detailed evaluation of 43 relevant articles. There were 7 RCTs and 1 retrospective study. Geographically, most of the places where the study was involved in this review were from France, followed by the United States, Indonesia and India.

All the 8 studies provided demographic data from the subjects, such as age, gender, GCS score at arrival, head CT findings, mode of injury, and physiological parameters (e.g., plasma osmolarity, patient's vital sign and arterial blood gas values). Six studies reported the mean values of ICP decrease, 6 studies reported the mean values of MABP scores, 3 studies reported the mean values of CPP scores, 2 studies listed the mortality rates, and 3 studies reported the neurological outcomes based on the Glasgow outcome scale score within 3 months. Conclusions were also collected from each study (Table 2).

**Table 2:** Study characteristics.

Study, Publication year	Country	Study design	Study population (*n*)	Demographic data	Conclusion
Ahmad and Hanna,^13^ 2014	Indonesia	RCT	42	Age, gender, GCS score, CT findings, CVP, MABP	Half-molar HSL was as effective as 20% mannitol in producing brain relaxation, with better hemodynamic stability and gave significant increase in blood glucose level
Ichai et al.,^14^ 2009	France	RCT	34	Age, gender, GCS score, CT findings, MABP, CPP	Acute infusion of a sodium lactate-based hyperosmolar solution is effective in treating intracranial hypertension following TBI
Ichai et al.,^15^ 2013	France	RCT	60	Age, gender, GCS score, MABP, CPP	A 48-h infusion of SL decreased the occurrence of raised ICP episodes in patients with severe TBI, while reducing fluid and chloride balances. These findings suggest that SL solution could be considered as an alternative treatment to prevent raised ICP following severe TBI
Cottenceau et al.,^16^ 2011	France	RCT	56	Age, GCS score, mode of injury, CT findings	mannitol was as effective as Hypertonic Saline in decreasing ICP in TBI patients
Francony et al.,^4^ 2008	France	RCT	20	Age, GCS score, body weight	A single equimolar infusion of 20% mannitol is as effective as 7.45% HSS in decreasing ICP in patients with brain injury
Jagannatha et al.,^17^ 2016	India	RCT	38	Age, gender, GCS score, mode of injury, CT findings	When used in equiosmolar doses, reduction in ICP with hypertonic saline is comparable to that with 20% mannitol in patients with severe TBI in the first 6 days after the injury. A steeper ICP reduction in response to hypertonic saline does not seem to confer any additional benefit in terms of overall ICP control after TBI
Kerwin et al.,^18^ 2009	USA	Retrospective	22	Age, gender, SBP/HR/RR at admission	Hypertonic saline is more efficacious than mannitol in reducing ICP
Oddo et al.,^19^ 2015	USA	RCT	12	Age, gender, GCS score, injury type	In patients with severe TBI and elevated ICP refractory to previous mannitol treatment, hypertonic saline administered as second tier therapy is associated with a significant increase in brain oxygenation, and improved cerebral and systemic hemodynamics.

RCT: randomized control trials; GCS: Glasgow coma scale; CVP: central venous pressure; MABP: mean arterial blood pressure; CPP: cerebral perfusion pressure; SBP: systolic blood pressure; HR: heart rate; RR: respiration rate; HSL: ; TBI: traumatic brain injury; ICP: intracranial pressure; HSS:.

After collecting data, we appraised the studies according to the evidence levels using data evaluation (7 RCTs and 1 retrospective study). Each research study had a random and good allocation which had been assessed as having good attrition values and reported bias values.

### Study bias risk assessment

Research quality was assessed by bias value which includes sequence generation (selection bias), allocation concealment (selection bias), blinding of participants and personnel (performance and detection bias), incomplete outcome data (attrition bias), selective reporting (reporting bias) and other bias sources (Table 3).

**Table 3:** Assessment of bias value.

Included studies	Random sequence generation?	Allocation concealment?	Incomplete outcome data (attrition bias)?	Selective reporting (reporting bias)?
Ahmad and Hanna,^13^ 2014	+	+	+	+
Ichai et al.,^14^ 2009	+	+	+	+
Ichai et al.,^15^ 2013	+	+	+	+
Cottenceau et al.,^16^ 2011	+	+	+	+
Francony et al.,^4^ 2008	+	+	+	+
Jagannatha et al.,^17^ 2016	+	+	+	+
Kerwin et al.,^18^ 2009	+	+	+	+
Oddo et al.,^19^ 2015	+	+	+	+

Note：“+” means yes.

### Therapeutic effects

From the results of data analysis, the two treatment groups of mannitol and half-molar sodium lactate did not show any significant differences.

#### ICP

**Fig. 2:**
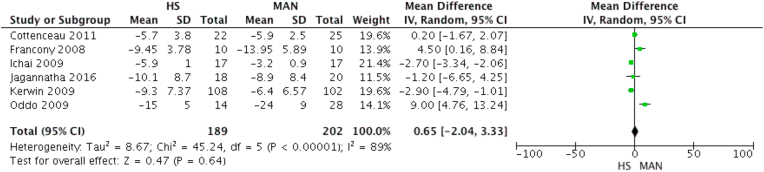
Effect of ICP decreasing.

The mannitol group was able to control ICP 0.65 times better than the half-molar sodium lactate group (MD 0.65; *p* = 0.64) (Fig. 2).

#### Hemodynamics

**Fig. 3:**
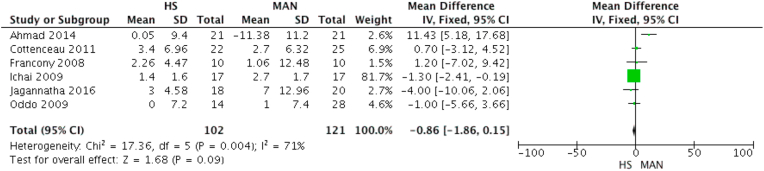
Effect on mean arterial pressure.

In the effect of hemodynamics described by the MABP parameter, there was no significant difference either. It was obtained that the half-molar sodium lactate group could maintain a MABP level 0.86 times better than the mannitol group (MD 0.86; *p* = 0.09) (Fig. 3).

#### CPP

**Fig. 4:**
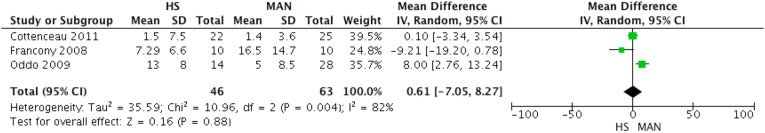
Effect on cerebral perfusion pressure.

As for the CPP parameter, it was found that the mannitol group was 0.61 times better at increasing CPP, compared to the half-molar sodium lactate group (MD 0.61; *p* = 0.88) (Fig. 4).

## Discussion

This review aims to compare the use of half-molar sodium lactate with mannitol for brain edema in severe TBI cases based on both effectiveness and safety aspects. Nowadays, mannitol is the first choice of standard osmotherapy for intracranial hypertension.^20^, ^21^, ^22^

### Effectiveness

Ahmad and Hanna^13^ in 2014 reported that half-molar sodium lactate and mannitol has similar effectiveness in controlling ICP and brain relaxation, but hyperosmolar lactate is superior in maintaining the hemodynamic stability, with an adverse effect of increased blood glucose level. MABP in half-molar sodium lactate group and mannitol group was not significantly different in the first 30 min; but in the longer duration (>30 min), half-molar sodium lactate group showed a more stable result.

Ichai et al.^14^ in 2009 reported that half-molar sodium lactate is safer and more effective compared with mannitol in decreasing ICP during an episode of intracranial hypertension among acute brain injury patients. Hyperosmolar lactate maintained a lower ICP for a longer period than mannitol did. The authors used the level of ICP as an independent outcome to describe the condition of episode of intracranial hypertension. At 30 min, there were no significant differences of ICP decreases between half-molar sodium lactate and mannitol groups. Nevertheless, in the longer duration (45 min), there was a significant increase of blood glucose in half-molar sodium lactate group (3.8% ± 1.3%, *p* < 0.01), while in mannitol group plasma glucose was not affected. As for the parameter of CPP, no significant differences was revealed at 30 min between two groups. However, in the longer period, the half-molar sodium lactate group showed a significant improvement.

In 2013, Ichai et al.^15^ conducted a research to find out the role of half-molar sodium lactate as a preventive therapy of brain edema in severe TBI patients. This research was a double-blind RCT on 60 patients with severe TBIs, on whom ICP monitor has already been placed. Parameters used were the same as those used in the previous research, which were ICP value, half-molar sodium lactate administered intravenously at rate of 0.5 mL/kg/h during 48 h after the trauma compared with control group which received isotonic saline. Result showed that half-molar sodium lactate group could significantly decrease the episode of increased ICP (23 episodes) compared with the control group (53 episodes).

Cottenceau et al.^16^ in 2004 did a research which compared the effect of hypertonic saline and that of mannitol for severe TBI. This RCT concluded that hypertonic saline is as effective as mannitol in lowering ICP, but it is better than mannitol in terms of hemodynamic stability. ICP (mmHg) was lower in hypertonic saline group than in mannitol group at 30 min after administration (12.2 ± 6.1 *vs.* 10.5 ± 6.8). However, at 120 min, there was no significant difference between the two groups (13.9 ± 7.8 *vs.* 13.6 ± 7.5). The baseline ICP (mmHg) in hypertonic saline and mannitol groups were 17.9 ± 9.9 and 16.3 ± 9.3 respectively. Similar effect was seen in mean arterial pressure (MAP) (mmHg) of the subjects at 30 min after infusion (91.2 ± 10.2 *vs.* 87.4 ± 11.6). Nevertheless, at 120 min, no significant difference was seen between the two groups, with the baseline MAP (mmHg) of hypertonic saline and mannitol group being 90.6 ± 12.6 and 87.6 ± 12.2, respectively.

Interestingly, Francony et al.^4^ reported that mannitol was better than hypertonic saline in terms of hemodynamic stability, as seen in MAP parameter (2% ± 9% *vs.* −5% ± 7%). CPP was reported to be better in mannitol group than in hypertonic saline group (21% ± 23% *vs*. 7% ± 11%). The author concluded that hypertonic saline is as effective as mannitol as an ICP-lowering therapy.

Likewise, Jagannatha et al.^17^ found that hypertonic saline is as effective as mannitol in lowering ICP (mmHg) (−10.1 ± 8.7 *vs.* 8.9 ± 8.4). The two groups were followed up until 6 days, but no significant difference was revealed.

A study by Kerwin et al.^18^ is the only retrospective study which compared the effectivity of hypertonic saline with mannitol in 20 severe TBI patients. Patients in hypertonic saline group had a higher baseline ICP (mmHg) than the mannitol group (30.7 ± 7.94 *vs.* 28.3 ± 8.07). This study concluded that hypertonic saline is more effective than mannitol in lowering ICP (mmHg) (9.3 ± 7.37 *vs.* 6.4 ± 6.57).

Oddo et al.,^19^ through their study on 12 severe TBI patients on whom ICP monitor had been placed, reported a different finding. All their subjects were initially given mannitol as the first line therapy, hypertonic saline was used as second-line therapy, hypertonic saline was given only if ICP remained at > 20 mmHg for more than 10 min despite the initial management or MAP dropped to ≤ 90 mmHg. Hypertonic saline was not given to patient with chronic hyponatremia, diabetes insipidus, heart failure, or central venous pressure > 15 mmHg. The authors concluded that hypertonic saline significantly improved CPP (mmHg) (76 ± 17 *vs.* 65 ± 19), and lowered ICP (mmHg) (15 ± 5 *vs.* 24 ± 9). Hemodynamic status in the hypertonic saline group was also reported to be more stable than in the mannitol group. The baseline ICP (mmHg) of this study subjects was 27 ± 8 *vs.* 29 ± 8, while the baseline CPP (mmHg) was 63 ± 15 *vs.* 60 ± 17.

Half-molar sodium lactate is as effective as mannitol in lowering ICP during the early phase of severe TBI. That being said, during the later period, half-molar sodium lactate is more effective than mannitol. Half-molar sodium lactate is also more effective to prevent the episode of ICP surge or intracranial hypertension than mannitol. Besides, half-molar sodium lactate seems better than mannitol in improving CPP and maintaining hemodynamic stability.

### Effects on biochemical parameter

#### Sodium serum

Cottenceau et al.^16^ found that there was a significant increase in serum sodium level (mmol/L) in the half-molar sodium lactate group compared to the mannitol group (148.3 ± 5.2 *vs.* 139.1 ± 4.1), with baseline (144.2 ± 5.1 *vs.* 141.3 ± 5.1).

Jagannatha et al.^17^ reported that there were no significant differences in serum sodium levels (mmol/L) between the half-molar sodium lactate group and the mannitol group (144 ± 11 *vs.* 140 ± 8, with the second baseline group 144 ± 4.

Kerwin et al.^18^ reported an increase in serum sodium in the half-molar sodium lactate (MD 1.9 mmol/L) group, with a maximal increase of 11 mmol/L.

Oddo et al.^19^ reported that in the half-molar sodium lactate group there was a significant increase in serum sodium (mmol/L) compared to the mannitol group (141 ± 6 before administration *vs.* 149 ± 6 after administration). In his study, 3 patients had hypernatremia (serum sodium> 155 mmol/L).

#### Serum osmolality

Ichai et al.^14^ reported a significant increase in the serum osmolality levels in the mannitol group compared to the half-molar sodium lactate group ((3.06 ± 0.67) mmol/kg, *p* < 0.001 *vs.* (0.66 ± 0.95) mmol/kg, *p* = 0.491).

Francony et al.^4^ reported that there were no significant differences of serum osmolarity (mmol/kg) between the two treatment groups (297.8 ± 13.1 *vs.* 298.9 ± 11.1), with baseline 292 ± 13 *vs.* 296 ± 11. Jagannatha et al.^17^ suggested that there was no significant difference in serum osmolarity (mmol/kg) between the two treatment groups (297 ± 22 *vs.* 290 ± 6), with baseline 302 ± 10 *vs.* 299 ± 10. Kerwin et al.^18^ obtained an increase in serum osmolarity in the half-molar sodium lactate (MD 2.8 mmol/kg) group, with a maximum increase of 27 mmol/kg. Oddo et al.^19^ reported that there was no significant difference in serum osmolality effects between the two treatment groups.

#### Blood glucose

Ahmad et al.^13^ reported a significant increase in blood glucose levels (mg/L) in the half-molar sodium lactate group compared to the mannitol group (141.81 ± 19.09 *vs.* 122.71 ± 17.89, *p* = 0.027), with a very significant difference between pre- and post-administration of half-molar sodium lactate (17.95 ± 11.46, *p* = 0.001).

Ichai et al.^14^ found that there was a high increase in blood glucose levels (mg/L) in the half-molar sodium lactate group (3.8% ± 1.3%, *p* < 0.01). Somewhat different, Jagannatha et al.^17^ reported that the mannitol group had higher blood glucose levels than the half-molar sodium lactate group (157 ± 39 *vs.* 126 ± 30), with baseline (173 ± 65 *vs.* 166 ± 33).

### Conclusion

Half-molar sodium lactate is as effective as mannitol in reducing ICP in the early phase of brain injury, but half-molar sodium lactate is superior over a longer period than mannitol. Moreover, it was concluded that half-molar sodium lactate can prevent the occurrence of episodes of intracranial hypertension, has a more stable effect on hemodynamics, and better brain tissue perfusion than the mannitol group. However, given that the administration of half-molar sodium lactate causes an increase in serum sodium, it is only safe to use in patients with serum sodium levels <150 mmol/L and osmolarity level of <310 mmol/kg.

## Funding

Nil.

## Ethical statement

Not applicable because this is a meta-analysis.

## Declaration of competing interest

The authors declared that no existing competing interests.
